# Development and validation of a nomogram prediction model for perioperative deep vein thrombosis risk in arthroplasty: a retrospective study

**DOI:** 10.3389/fmed.2025.1528154

**Published:** 2025-05-22

**Authors:** Wenming Yang, Qitai Lin, Zehao Li, Chuanjie Shan, Xiaoyu Cheng, Yugang Xing, Yongsheng Ma, Yang Liu, Meiming Li, Ruifeng Liang, Wangping Duan, Pengcui Li, Xiaochun Wei

**Affiliations:** ^1^Academy of Medical Sciences, Shanxi Medical University, Taiyuan, China; ^2^Department of Orthopaedics, Second Hospital of Shanxi Medical University, Taiyuan, China; ^3^Shanxi Key Laboratory of Bone and Soft Tissue Injury Repair, Taiyuan, China; ^4^Department of Environmental Health, School of Public Health, Shanxi Medical University, Taiyuan, China

**Keywords:** arthroplasty, deep vein thrombosis, risk factor, thromboelastography, nomogram

## Abstract

**Background:**

Perioperative monitoring thrombosis has become more crucial due to the rising demand for arthroplasty and shorter hospital stays. We aimed to comprehensively explore immune-inflammatory and hypercoagulable states during perioperative periods patients undergoing arthroplasty to identify the risk factors for early postoperative deep vein thrombosis (DVT) and construct a nomogram prediction model for postoperative DVT.

**Methods:**

Electronic medical records of 841 patients who underwent primary arthroplasty at a single institution were retrospectively reviewed. Patients’ demographic and perioperative laboratory data were collected and divided into training (73.8%) and validation sets (26.2%) based on order of procedure date. Variables were screened from the training set using the Least Absolute Shrinkage and Selection Operator (LASSO) regression; a nomogram was constructed after multivariate logistic regression. The validation set was used to evaluate its discriminatory capacity and efficacy. The model’s performance was evaluated through the Brier score, receiver operating characteristic curves, area under the curve (AUC), calibration curves, decision curve analysis (DCA), and clinical impact curves (CIC).

**Results:**

We found an asymptomatic DVT incidence of 27.5% (231/841) on postoperative day three and identified seven predictors: age, chronic heart failure, stroke, tourniquet, postoperative monocyte-to-lymphocyte ratio, and postoperative alpha and D-dimer levels. The predictive model yielded an AUC of 0.737 (95% CI, 0.6933–0.7785), with an external validation AUC of 0.683 (95% CI, 0.6139–0.7716). The Brier score was 0.176, indicating the model’s strong robustness in predicting perioperative DVT incidence in arthroplasty. Clinical impact and decision curve analysis revealed that using the proposed nomogram for prediction yielded a net benefit for threshold probabilities of 10–70%.

**Conclusion:**

Our risk prediction model demonstrated reasonable discriminative capacity for predicting perioperative DVT risk in arthroplasty. This model may help increase the clinical benefits for patients by promptly identifying high-risk individuals early postoperatively.

## Introduction

Lower limb arthroplasty involving surgical hip or knee joint replacement is a common treatment for advanced hip or knee joint diseases. The increasing prevalence of osteoarthritis due to aging and obesity as well as considerable improvements in postoperative quality of life are key reasons for the rapidly increasing implementation of this procedure (1). Arthroplasty is anticipated to become the most common elective surgery in the United States (2). Similarly, the volume of arthroplasty procedures in China is growing at a rate of 20% annually (3).

Despite its benefits, arthroplasty involves a substantial risk of deep vein thrombosis (DVT). In the absence of pharmacological prophylaxis, the perioperative incidence of all DVTs was 16.8–46.8% in several studies based on an Asian population (4–6). Many DVTs are isolated distal DVTs, and about 10–20% of isolated distal DVTs may extend to proximal DVTs (7), with 4–15% causing symptoms or pulmonary embolism (8).

The incidence of DVT during the perioperative period following major orthopedic surgery is highest within the first 24 h (9), with a peak occurrence of symptomatic thrombosis observed at one week postoperatively (10, 11). To balance the risk between postoperative bleeding and DVT, mechanical prophylaxis is typically used alone during the initial 12–24 h postoperatively, leaving a gap for pharmacologic prophylaxis (9). The average length of hospitalization after arthroplasty is three days (12). Relying solely on early signs or symptoms of DVT is an unreliable strategy for preventing clinically significant thromboembolic events (13). Early and accurate prediction of the risk of perioperative DVT is crucial for preventing severe complications.

Recently, the association between immune-inflammatory and venous thrombosis has been recognized (14). Furthermore, an interaction between inflammatory and hypercoagulable states has been recognized (15, 16). Thromboelastography (TEG), a method for assessing coagulation dynamics using whole blood, facilitates dynamic monitoring of the entire clotting reaction and exhibits relatively high sensitivity in detecting hypercoagulable states (17). Novel inflammatory markers derived from hematological reports, such as the monocyte-to-lymphocyte ratio (MLR), neutrophil-to-lymphocyte ratio (NLR), platelet-to-lymphocyte ratio, platelet-to-neutrophil ratio, systemic immune-inflammation index (SII), systemic immune response index (SIRI), and aggregate index of systemic inflammation (AISI), serve as a cost-effective and readily accessible indicator of overall inflammation levels in the body (18). These peripheral blood-derived indices have been increasingly employed in the study of complications in large-scale orthopedic surgeries, demonstrating promising predictive values (19–23).

Despite extensive research on the risk factors for DVT, the focus has primarily been on symptomatic DVT. To our knowledge, reliable predictive models for asymptomatic DVT during the perioperative periods are currently lacking. Therefore, in this study, we aimed to integrate the perioperative immune–inflammatory status and hypercoagulable state before anticoagulation intervention to identify risk factors for perioperative DVT in patients undergoing arthroplasty. We sought to create and validate a nomogram prediction model to estimate the risk of early postoperative DVT in a simple and accurate manner.

## Patients/methods

### Patients

Medical records of hospitalized patients from July 1, 2022, to October 30, 2023, at the Second Hospital of Shanxi Medical University were collected. The inclusion criteria for patients were age ≥18 years, primary arthroplasty, and surgical procedures following the ICD-9-CM criteria for joint arthroplasty, which involve hemiarthroplasty, total knee arthroplasty, unicompartmental knee arthroplasty, and total hip arthroplasty (24). The exclusion criteria for patients were as follows: (1) preoperative DVT; (2) malignant tumors, severe pneumonia, pathological fractures, or other diseases that could affect the occurrence of DVT; (3) recent history of surgery or trauma; (4) incomplete examination items; and (5) preoperative use of anticoagulant drugs.

According to the requirements for establishing a clinical prediction model (25), the previous incidence of postoperative DVT in arthroplasty was approximately 0.40 (5, 6), and the c-statistic of the prediction model was 0.80 (26–28). Additionally, 20 predictive variables were scheduled for collection. The developmental dataset’s minimum sample size was calculated to be 609 using “pmsampsize.” Data from June 2022 to July 2023 were used as the training dataset to develop the model, and data from August to October 2023 were used as the validation dataset.

### Ethical approval

This single-center, retrospective observational study was conducted at a tertiary teaching hospital in China and was approved by the Institutional Ethics Committee of the Second Hospital of Shanxi Medical University (No: 2023KYNO. [173]). The study was performed in accordance with the Declaration of Helsinki. As this was a retrospective study, and data were analyzed anonymously, informed consent was waived by the committee.

### Assessment of DVT

According to institutional surgical procedures, patients scheduled for arthroplasty were required to undergo a bilateral lower limb complete duplex ultrasound (CDUS) (29) examination both preoperatively and on postoperative day three. The CDUS examination was performed by two experienced radiologists, and the results were reviewed by another senior radiologist. The criteria for diagnosing DVT included inadequate flow to the foot and calf determined via compression procedures, lumen blocking or filling defects, and vein absence or non-compressibility (30). The patients were separated into two groups based on the CDUS results (DVT and non-DVT), with the DVT group including both proximal DVT and isolated distal DVT (8).

### Surgery and thromboprophylaxis

All procedures were performed in the same institution and by eight senior surgeons. The mean operation time was 94 min. Knee replacement surgery typically involves the use of a tourniquet to manage bleeding, which is released after wound closure. Only a few patients underwent inflation of the pneumatic tourniquet before prosthesis installation, followed by immediate deflation after bone cement fixation. Prior to wound closure, a combination treatment comprising tranexamic acid, ropivacaine, dexamethasone, and adrenaline mixture was injected around the joint to facilitate hemostasis and alleviate pain. In contrast, hip replacement surgery did not use pneumatic tourniquets or bone cement, with only tranexamic acid injected into the joint space to control bleeding during suturing. We actively implemented postoperative physical prophylaxis, wearing elastic socks, and ankle pump exercise, and encouraged patients to move as early as possible. For most patients, partial weight loading was allowed 24–48 h after surgery. Drug prophylaxis or treatment was administered after postoperative CDUS evaluation.

### Data collection

The following data were gathered and examined from patient medical records, including demographic data (age, sex, body mass index [BMI], tobacco and alcohol use, surgery history, and blood transfusion history), comorbidities (hypertension, diabetes, hyperlipidemia, stroke, varicose veins, chronic heart failure), which were reported by patients themselves or their guardians at the time of admission. Surgical information (diagnosis, surgery, and hospitalization), ultrasound findings (presence of DVT and thrombus location), and laboratory findings before and after surgery (complete blood count, conventional coagulation tests, TEG, C-reactive protein, and erythrocyte sedimentation rate) were also obtained and assessed. Surgical patients followed a standardized blood sampling procedure. Peripheral venous blood samples are routinely collected before and on the morning of the first day after surgery for laboratory tests, namely laboratory results within 24 h after surgery.

### Statistical analysis

Owing to the non-uniformity in dimer units, the D-dimer units were adjusted through multiplication with a factor of “2” to obtain equivalent fibrinogen equivalent units (FEUs) prior to analysis (31). Data analysis was performed using SPSS 29.0 (IBM Corp., Armonk, NY, USA) and R software (R Software for Statistical Computing, Vienna, Austria). Continuous variables with normal distribution are presented as mean ± standard deviation and otherwise as median and interquartile range. Between-group differences were compared using the independent t-test and Mann–Whitney U test. Ordinal and categorical variables are represented by numbers (*n*) and percentages (%), and group differences were analyzed using the chi-squared test or Fisher’s exact test.

Variable selection was performed using the least absolute shrinkage and selection operator (LASSO) regression. The optimal lambda value, lambda.1se, was determined through cross-validation to ensure model simplicity and robustness in selecting risk factors. Subsequently, multivariable logistic regression analysis was conducted based on these factors to establish a predictive model for postoperative DVT. The model’s performance and various risk factors were analyzed using receiver operating characteristic (ROC) curves and the area under the curve (AUC). The model’s performance was comprehensively assessed using the Brier score, calibration curve, decision curve analysis (DCA), and the clinical impact curve (CIC). Finally, the model was validated using a validation set. Statistical significance was set at *p* < 0.05.

## Results

### Comparison between training and validation sets

**Figure 1:**
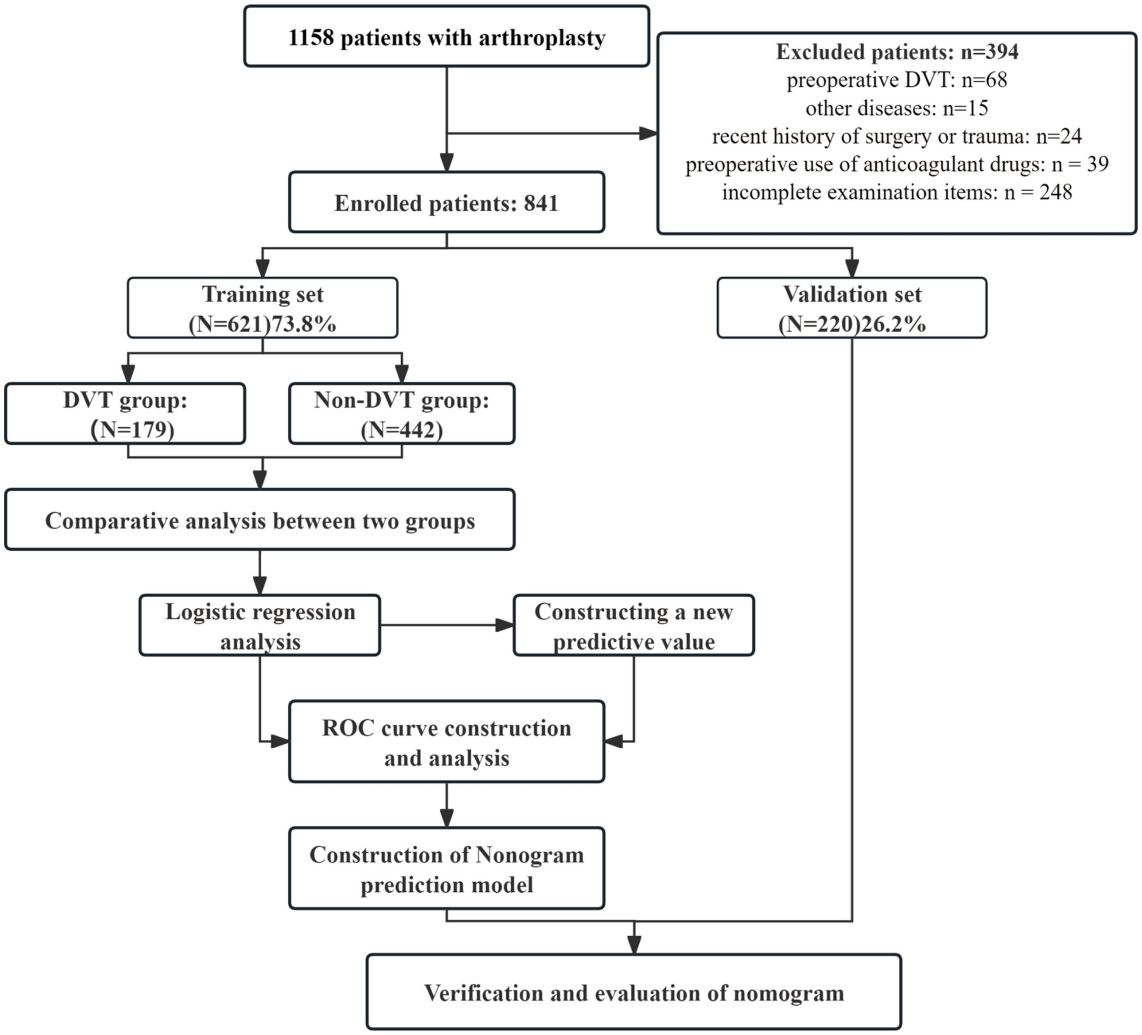
Patient selection flowchart.

A total of 841 patients (median age, 66 years; 575 females [68.4%]) underwent hip and knee replacement surgeries. There were 231 patients (27.5%) diagnosed with DVT using CDUS on postoperative day three, of which seven had proximal DVT, accounting for only 0.8%. Symptomatic DVT was not yet seen. According to the flowchart shown in Figure 1, 621 (73.8%) of 841 patients were included in the training dataset, whereas 220 (26.2%) were included in the validation dataset. The training set’s sample size satisfies the requirements for the development of models. Table 1 presents the demographic and clinical characteristics of the inpatients comprising the training set and the validation set. The incidence of DVT (28.8% vs. 23.6%, *p* = 0.138) did not significantly differ between the groups. There was a balanced comparability of the baseline characteristics between the datasets.

**Table 1:** Demographic and clinical characteristics of study participants.

Variable	Training set (*n* = 621)	Validation set (*n* = 220)	All patients (*n* = 841)	*p* value
Postoperative DVT, *n* (%)	179 (28.8)	52 (23.6)	231 (27.5)	0.138
Sex (Female), *n* (%)	415 (66.8)	160 (72.7)	575 (68.4)	0.106
Age (years), (median [IQR])	66 [60, 70]	67 [60, 72]	66 [60, 71]	0.094
BMI (≥ 28 kg/m^2^), *n* (%)	167 (26.9)	53 (24.1)	220 (26.2)	0.394
Length of stay (days), (median [IQR])	8 [7, 9]	7 [7, 9]	7 [7, 9]	0.092
Smoker, *n* (%)	87 (14.0)	24 (10.9)	111 (13.2)	0.243
Drinker, *n* (%)	75 (12.1)	22 (10.0)	97 (11.5)	0.407
Hypertension, *n* (%)	296 (47.7)	101 (45.9)	397 (47.2)	0.654
Diabetes, *n* (%)	84 (13.5)	31 (14.1)	115 (13.7)	0.834
Chronic heart failure, *n* (%)	60 (9.7)	23 (10.5)	83 (9.9)	0.735
Hyperlipidemia, *n* (%)	9 (1.4)	5 (2.3)	14 (1.7)	0.376
Stroke, *n* (%)	28 (4.5)	16 (7.3)	44 (5.2)	0.114
Varicose vein, *n* (%)	7 (1.1)	2 (0.9)	9 (1.1)	1.000
History of blood transfusion, *n* (%)	16 (2.6)	9 (4.1)	25 (3.0)	0.256
Previous surgery, *n* (%)	267 (43.0)	107 (48.6)	374 (44.5)	0.148
Duration of surgery (min), (median [IQR])	95 [80, 110]	90 [80, 110]	93 [80, 110]	0.083
Mode of operation, *n* (%)				0.407
UKA	112 (18.0)	48 (21.8)	160 (19.0)	
TKA	351 (56.5)	112 (50.9)	463 (55.1)	
THA	140 (22.5)	51 (23.2)	191 (22.7)	
HA	18 (2.9)	9 (4.1)	27 (3.2)	
Protopathy, *n* (%)				0.806
OA	458 (73.8)	166 (75.5)	624 (74.2)	
RA	23 (3.7)	7 (3.2)	30 (3.6)	
AVN	75 (12.1)	21 (9.5)	96 (11.4)	
DDH	26 (4.2)	9 (4.1)	35 (4.2)	
FNF	39 (6.3)	17 (7.7)	56 (6.7)	
Spinal anesthesia, *n* (%)	581 (93.6)	216 (98.2)	797 (94.8)	0.008*
Air pressure therapy, *n* (%)	185 (29.8)	57 (25.9)	242 (28.8)	0.274
Intraoperative blood loss (≥200 mL), n (%)	161 (25.9)	32 (14.5)	193 (22.9)	0.001*
Tourniquet, *n* (%)	440 (70.9)	160 (72.7)	600 (71.3)	0.597
Intraoperative blood transfusion, *n* (%)	76 (12.2)	17 (7.7)	93 (11.1)	0.067
Drainage tube, *n* (%)	156 (25.1)	46 (20.9)	202 (24.0)	0.209

AVN, Avascular necrosis; BMI, Body mass index; DDH, Developmental dysplasia of the hip; FNF, Fracture of neck of femur; IQR, Interquartile range; OA, Osteoarthritis; DVT, Deep venous thrombosis; HA, Hemiarthroplasty; RA, Rheumatic arthritis; THA, Total hip arthroplasty; TKA, Total knee arthroplasty; UKA, Unicompartmental knee arthroplasty. ^*^Significant difference (*p* < 0.05).

### Clinical indicator analysis

Univariate analysis of the training set (Table 2) revealed the following parameters to be significantly associated with postoperative DVT: age, sex, smoking, hypertension, diabetes, chronic heart failure, stroke, surgery time, surgical mode, protopathy, intraoperative blood loss, tourniquet use, Preoperative ESR, postoperative NLR (post-NLR), postoperative MLR (post-MLR), postoperative SIRI (post-SIRI), postoperative AISI (post-AISI), postoperative k value (post-k), postoperative alpha (post-*α*) levels, and postoperative D-dimer (post-D-dimer) levels. When comparing the baseline characteristics of the non-DVT and DVT groups in all datasets (Supplementary Table 1), the results of the all-patient analysis were comparable to those of the training set. Analyzing baseline data for hip and knee subgroups in the training set reveals slight differences in some risk factors (Supplementary Table 2). Risk factors for knee replacement align with the validation set. However, the hip subgroup’s smaller sample size (*n* = 158) compared to the knee group (*n* = 463) could result in omitting some important variables when modeling separately.

**Table 2:** Univariate analysis of the non-DVT and DVT groups in the training set.

Variable	Non-DVT group (*n* = 442)	DVT group (*n* = 179)	All patients (*n* = 621)	*p* value
Sex (Female), *n* (%)	282 (63.8)	133 (74.3)	415 (66.8)	0.012*
Age (years), *n* (%)				<0.001*
< 60	115 (26.0)	20 (11.2)	135 (21.7)	
60–69	220 (49.8)	92 (51.4)	312 (50.2)	
≥ 70	107 (24.2)	67 (37.4)	174 (28.0)	
BMI (≥28 kg/m^2^), *n* (%)	119 (26.9)	48 (26.8)	167 (26.9)	0.978
Smoker, *n* (%)	71 (16.1)	16 (8.9)	87 (14.0)	0.021*
Drinker, *n* (%)	61 (13.8)	14 (7.8)	75 (12.1)	0.053
Hypertension, *n* (%)	197 (44.6)	99 (55.3)	296 (47.7)	0.015*
Diabetes, *n* (%)	51 (11.5)	33 (18.4)	84 (13.5)	0.023*
Chronic heart failure, *n* (%)	30 (6.8)	30 (16.8)	60 (9.7)	<0.001*
Stroke, *n* (%)	13 (2.9)	15 (8.4)	28 (4.5)	0.003*
Varicose vein, *n* (%)	5 (1.1)	2 (1.1)	7 (1.1)	0.988
Duration of surgery (min), (median [IQR])	95.00 [83.25, 112.75]	90.00 [75.00, 109.50]	95.00 [80.00, 110.00]	0.009*
Mode of operation, *n* (%)				<0.001*
UKA	84 (19.0)	28 (15.6)	112 (18.0)	
TKA	224 (50.7)	127 (70.9)	351 (56.5)	
THA	124 (28.1)	16 (8.9)	140 (22.5)	
HA	10 (2.3)	8 (4.5)	18 (2.9)	
Protopathy, *n* (%)				<0.001*
OA	305 (69.0)	153 (85.5)	458 (73.8)	
RA	20 (4.5)	3 (1.7)	23 (3.7)	
AVN	68 (15.4)	7 (3.9)	75 (12.1)	
DDH	18 (4.1)	8 (4.5)	26 (4.2)	
FNF	31 (7.0)	8 (4.5)	39 (6.3)	
Spinal anesthesia, *n* (%)	27 (6.1)	13 (7.3)	40 (6.4)	0.596
Air pressure therapy, *n* (%)	130 (29.4)	55 (30.7)	185 (29.8)	0.746
Intraoperative blood loss (≥200ml), n (%)	125 (28.3)	36 (20.1)	161 (25.9)	0.035*
Tourniquet, *n* (%)	288 (65.2)	152 (84.9)	440 (70.9)	<0.001*
Intraoperative blood transfusion, *n* (%)	56 (12.7)	20 (11.2)	76 (12.2)	0.606
Drainage tube, *n* (%)	106 (24.0)	50 (27.9)	156 (25.1)	0.304
Preoperative ESR (mm/H), (median [IQR])	14.00 [7.00, 21.00]	14.00 [8.00, 25.00]	14.00 [8.00, 22.00]	0.041*
Postoperative NLR, (median [IQR])	8.52 [6.33, 11.93]	9.35 [6.61, 13.17]	8.68 [6.43, 12.34]	0.047*
Postoperative PLR, (median [IQR])	204.43 [151.57, 269.62]	224.72 [159.64, 283.06]	207.83 [154.63, 275.82]	0.101
Postoperative MLR, (median [IQR])	0.63 [0.49, 0.84]	0.73 [0.51, 1.05]	0.65 [0.49, 0.87]	0.001*
Postoperative PNR, (median [IQR])	23.95 [19.07, 29.45]	23.80 [17.99, 29.19]	23.89 [18.80, 29.42]	0.422
Postoperative SII, (median [IQR])	1878.57 [1212.87, 2619.91]	1994.81 [1305.45, 2901.62]	1925.84 [1240.75, 2685.80]	0.111
Postoperative SIRI, (median [IQR])	5.55 [3.94, 8.30]	6.73 [4.43, 10.22]	5.74 [3.98, 8.77]	0.003*
Postoperative AISI, (median [IQR])	1208.93 [729.57, 2005.97]	1403.97 [889.66, 2211.59]	1250.47 [778.26, 2037.45]	0.013*
Postoperative R (min), (median [IQR])	5.80 [5.20, 6.40]	5.80 [5.20, 6.30]	5.80 [5.20, 6.40]	0.296
Postoperative K (min), (median [IQR])	1.30 [1.20, 1.60]	1.20 [1.10, 1.50]	1.30 [1.20, 1.60]	0.026*
Postoperative α, (median [IQR])	69.20 [66.53, 71.80]	70.00 [67.80, 72.30]	69.50 [66.80, 71.90]	0.019*
Postoperative MA (mm), (median [IQR])	64.15 [60.90, 67.30]	64.30 [61.40, 67.00]	64.20 [61.00, 67.20]	0.563
Postoperative CI, (median [IQR])	1.00 [0.20, 1.80]	1.10 [0.40, 1.70]	1.00 [0.20, 1.80]	0.166
Postoperative D-dimer (ug/ml FEU), (median [IQR])	2.75 [1.61, 5.52]	4.58 [2.15, 7.47]	3.14 [1.76, 6.22]	<0.001*

AVN, Avascular necrosis; BMI, Body mass index; DDH, Developmental dysplasia of the hip; FNF, Fracture of neck of femur; IQR, Interquartile range; OA, Osteoarthritis; DVT, Deep venous thrombosis; HA, Hemiarthroplasty; RA, Rheumatic arthritis; THA, Total hip arthroplasty; TKA, Total knee arthroplasty; UKA, Unicompartmental knee arthroplasty; FEU, Fibrinogen equivalent units; ESR, Erythrocyte sedimentation rate; NLR, Neutrophil-to-lymphocyte ratio; MLR, Monocyte-to-lymphocyte ratio; PLR, Platelet-to-lymphocyte ratio; PNR, Platelet-to-neutrophil ratio; SII, Systemic immune-inflammation index; SIRI, Systemic immune response index; AISI, Aggregation index of systemic inflammation; R, Reaction time; K, *k* value; α, Alpha angle; MA, Maximum amplitude; CI, Coagulation index; *Significant difference (*p* < 0.05).

### Screening for predictive factors

**Figure 2:**
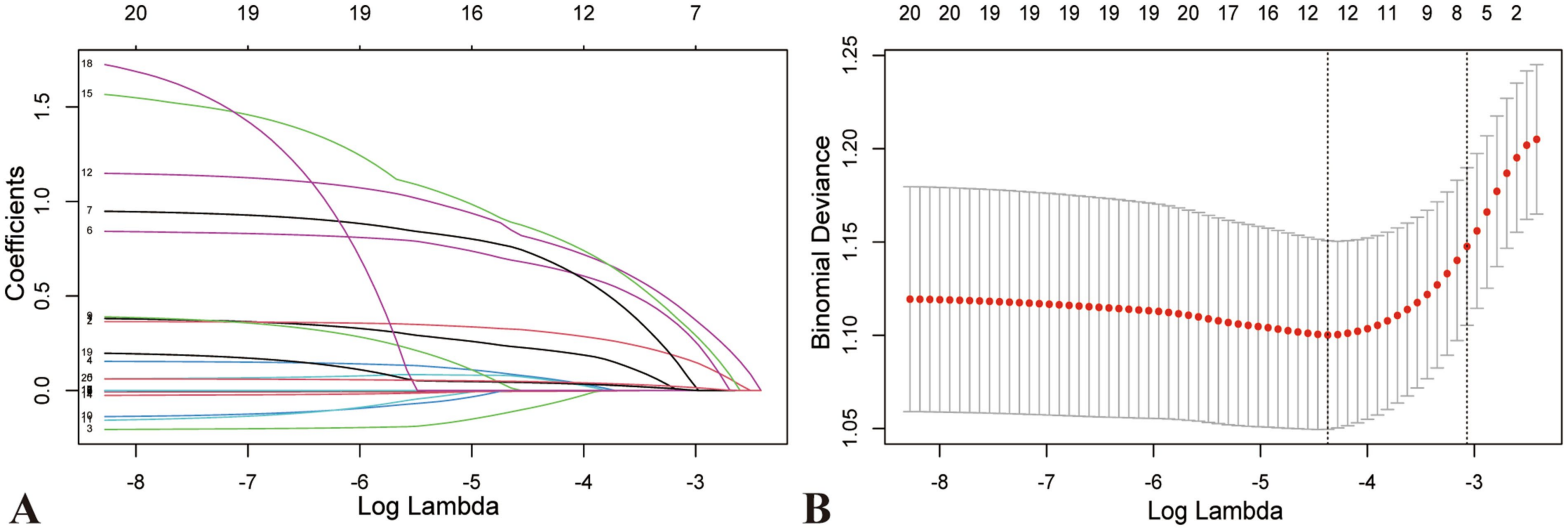
Characteristic variable screening based on LASSO regression analysis and Lasso regression parameter diagram. **(A)** Coefficient profile plot created against the log (lambda) sequence. **(B)** Lambda (adjustment parameter) was obtained by cross-validated LASSO regression. The right dotted line is Lambda at standard error (Lambda.1-SE). The left dotted line is Lambda at minimum error (Lambda.min). Our study selected the variables filtered by Lambda.1-SE, where seven non-zero coefficients were selected. LASSO, Least absolute shrinkage and selection operator; SE, standard error.

**Figure 3:**
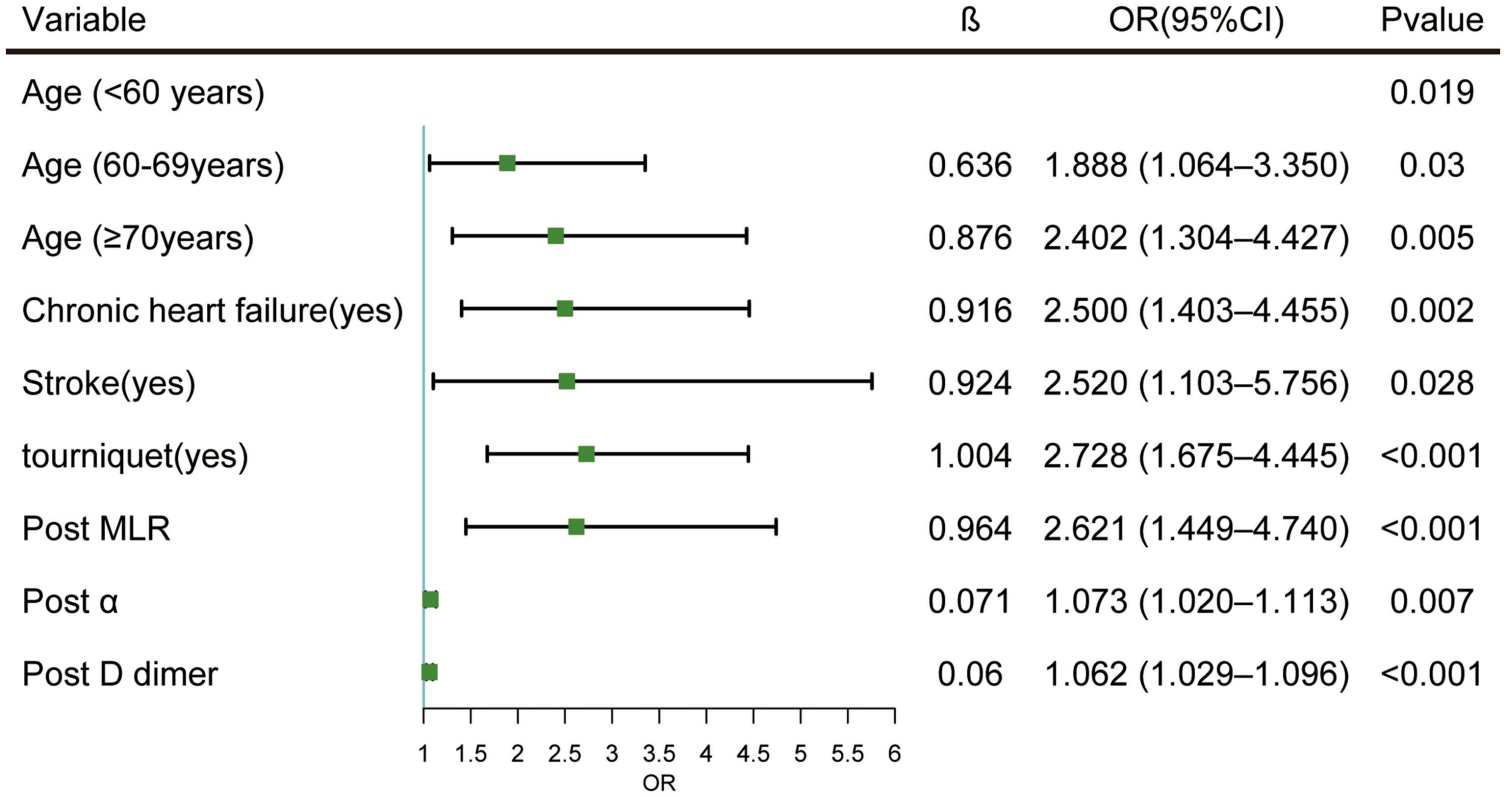
Multivariate logistic regression of screened variables in the development dataset. Cl, Confidence interval OR, Odds ratio.

Significant factors from the univariate analysis were included in the LASSO regression analysis for variable selection, and cross-validation was employed to determine the optimal lambda value (Figures 2A,B). Seven non-zero coefficient variables were selected as potential predictive factors, and the matching postoperative DVT prediction model was built via multifactorial logistic regression analysis based on the variables. Age (60–70, odds ratio [OR]: 1.481, *p* = 0.03; ≥70, OR: 2.447, *p* = 0.005), chronic heart failure (OR: 2.500, *p* = 0.002), stroke (OR: 2.520, *p* = 0.028), tourniquet (OR: 2.728, *p* < 0.001), post-D-dimer levels (OR: 1.062, *p* < 0.001), post-*α* levels (OR: 1.073, *p* = 0.007), and post-MLR (OR: 2.621, *p* < 0.001), demonstrated statistical significance as independent predictors of postoperative DVT (Figure 3).

### ROC curve construction and analysis

**Figure 4:**
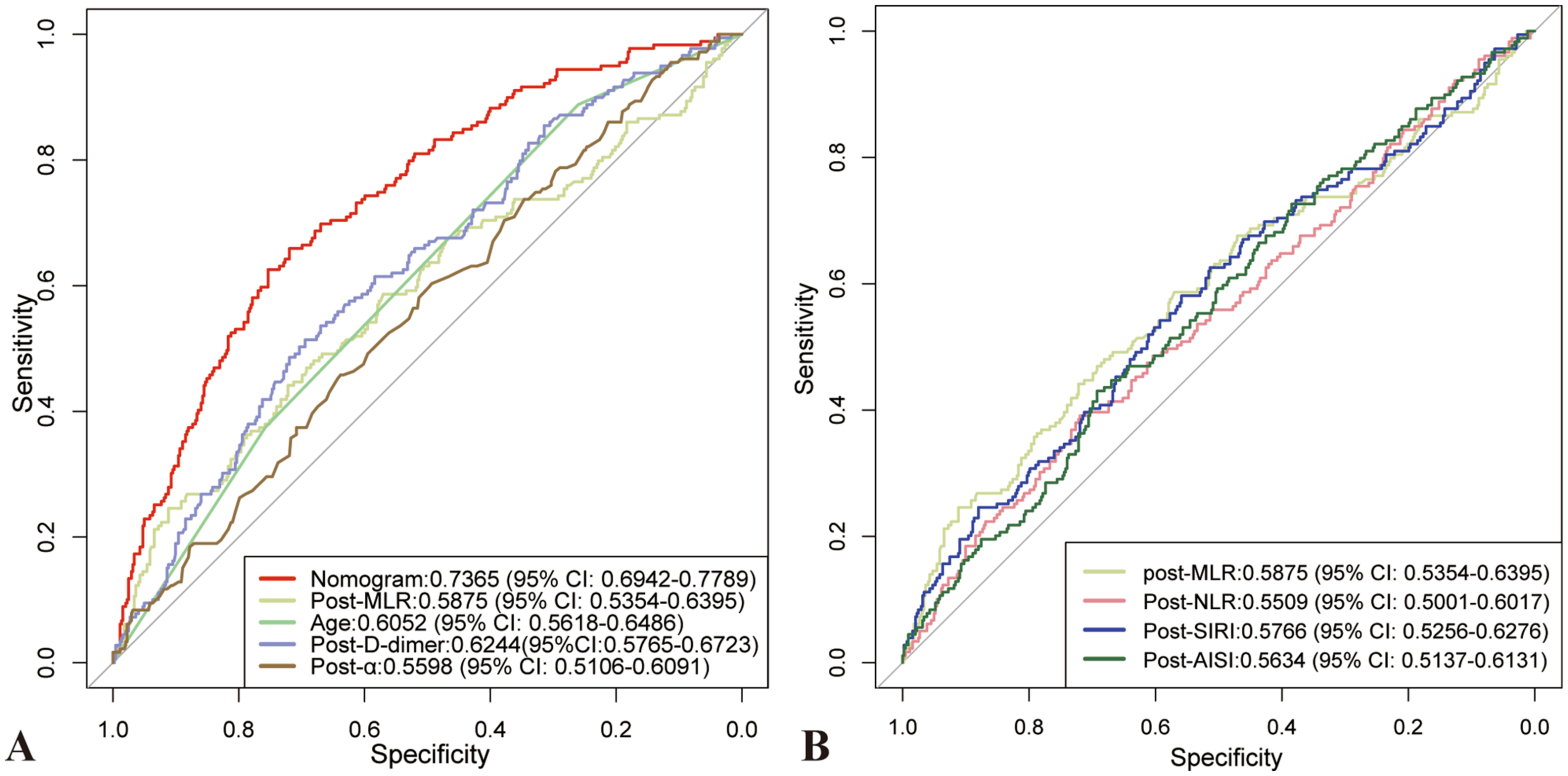
The ROC curves and AUC values for each risk factor in the models. **(A)** ROC curve and AUC for the predictive model and each risk factor in the model. **(B)** ROC curve and AUC values of blood immune-inflammatory markers. AUC, Area under the curve; ROC, Receiver operating characteristic.

Predictive models were constructed, and the ROC curves and AUC values for each risk factor in the models were plotted (Figure 4). The AUC for the predictive model was 0.7365 (95% confidence interval [CI], 0.6942–0.7789), with a cutoff value of 0.302, sensitivity of 71.9%, and specificity of 67%. The AUC values for post-MLR, age, post-D-dimer levels, and post-α were 0.5875, 0.6052, 0.6244, and 0.5598, respectively. Therefore, the predictive efficiency of this model was relatively high, with sensitivity and specificity superior to those of all individual independent risk factors. When developing ROC curves and calculating AUC values for novel inflammatory markers from hematological reports, postoperative MLR demonstrated greater predictive significance than NLR, AISI, and SIRI.

### Development and validation of nomogram

**Figure 5:**
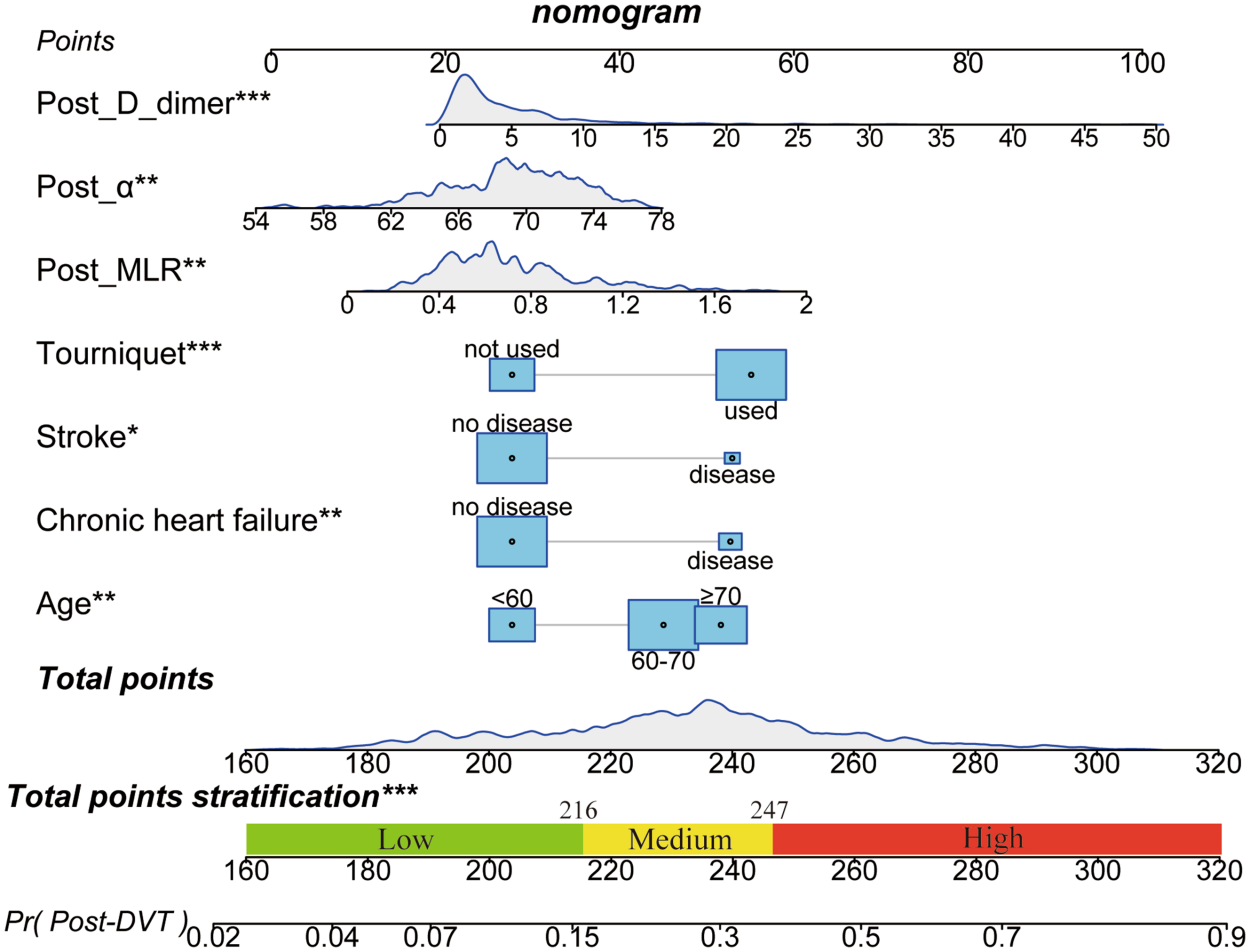
Nomogram for predicting postoperative DVT in patients with arthroplasty. The nomogram was developed using the seven significant influencing factors in the training set. The total points stratification in the nomogram prediction chart can distinguish the risk-level of patients for developing perioperative DVT. *p < 0.05, **p < 0.01, ***p < 0.001. DVT, Deep vein thrombosis.

**Figure 6:**
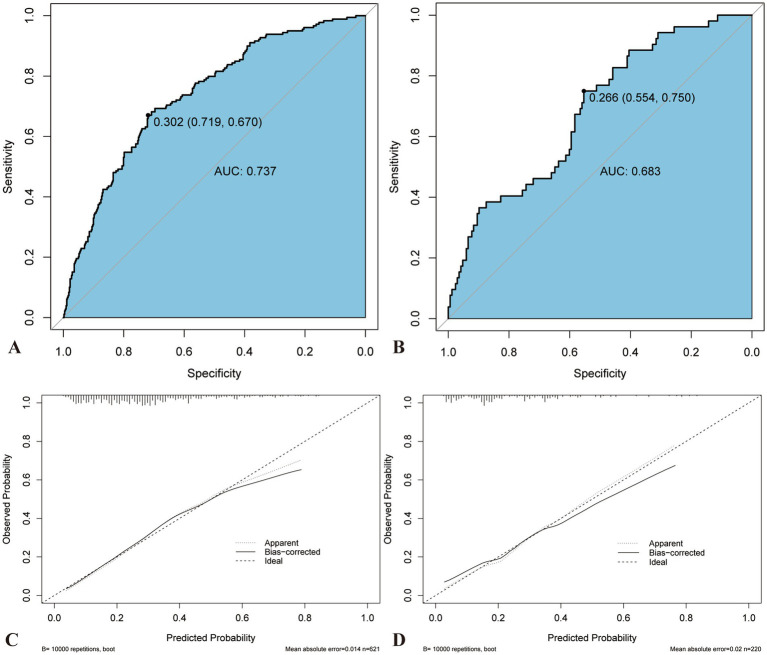
Comparison of AUC and calibration curve between training and validation sets. The ROC curve of nomogram in the training **(A)** and verification sets **(B)**. The prediction accuracy of nomogram was positively correlated with the AUC. The AUC values of nomogram in the training and verification sets were 0.739 and 0.693, respectively, indicating that the model has good discriminant ability. Calibration curve of the training **(C)** and verification sets **(D)**. AUC, Area under the curve; ROC, Receiver operating characteristics.

**Figure 7:**
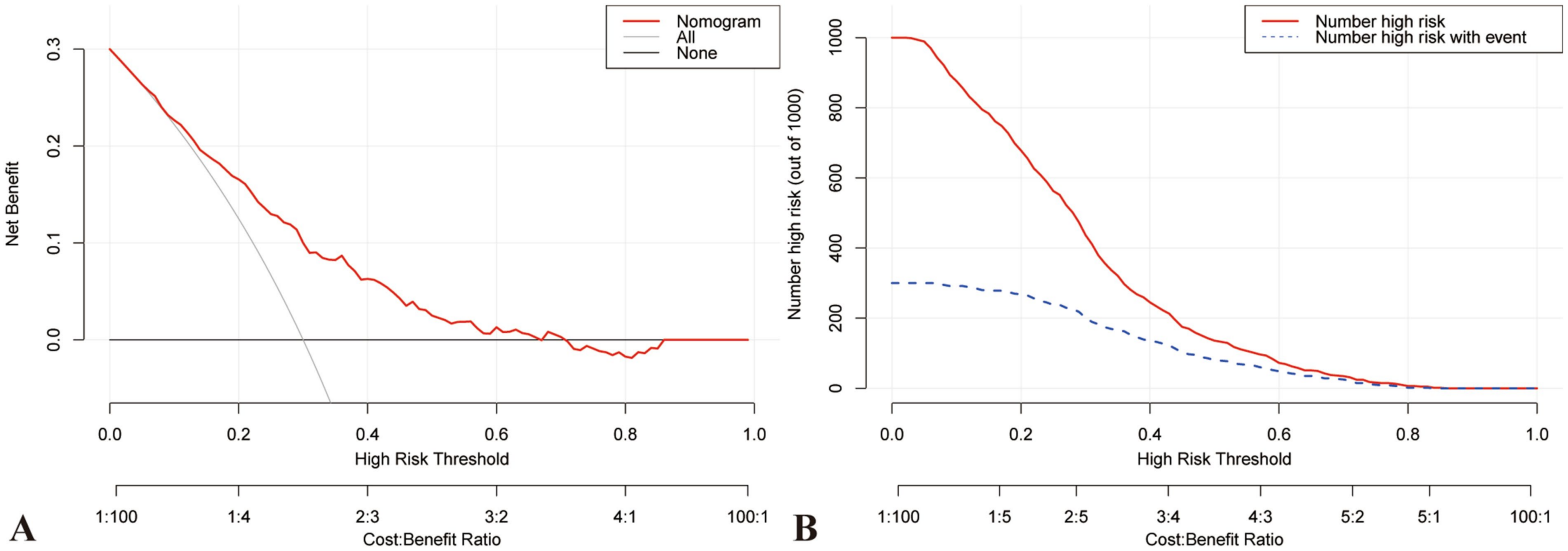
DCA and CIC curve for training set. **(A)** DCA demonstrates that the training model falls within the threshold probability range of 10–70%, and the decision curve lies above both the Null line and the all line, indicating its good clinical applicability. **(B)** CIC represents high-risk individuals identified by the model, along with the number of true positive individuals at various probability thresholds. This intuitive representation more accurately reflects the potential clinical net benefit of the predictive model. CIC, Clinical impact curve; DCA, Decision curve analysis.

A nomogram was constructed to visualize the risk of postoperative lower limb DVT in patients who underwent arthroplasty (Figure 5). In terms of the total score stratification, the risk of DVT gradually increased, indicating a certain degree of discrimination in the model. The Brier score was 0.1762 (<0.25), indicating the robustness of the model in predicting the incidence of perioperative DVT in arthroplasty. The Hosmer–Lemeshow goodness-of-fit test resulted in a χ^2^ value of 7.315 and a *p*-value of 0.503 (*p* > 0.05). The training set AUC (0.737, 95% CI, 0.6942–0.7789) and validation set AUC (0.683, 95% CI, 0.6010–0.7606) were calculated (Figure 6). The model was calibrated using the bootstrap method. The calibration curve of the training set closely resembled the ideal curve, indicating a high consistency between the predicted and actual occurrence rates of lower limb DVT in patients undergoing hip and knee arthroplasty. Despite a slight decrease in the model’s predictive ability in the validation set, it still demonstrated good discriminative ability and accuracy in identifying high-risk individuals. DCA showed that the nomogram risk prediction model provided more significant net benefits than the universal intervention and universal non-intervention strategies, supporting its high clinical applicability. The CIC, plotted using the nomogram, represents the number of high-risk individuals identified by the model and true-positive individuals at different threshold probabilities, reflecting the high net clinical benefit of the prediction model (Figure 7).

## Discussion

The decrease in the length of stay for arthroplasty has increased the need for enhanced perioperative monitoring (32). We hope that preemptive risk alerts can guide personalized anticoagulation before discharge. To our knowledge, this was the first study to develop a clinical prediction model for early postoperative DVT by combining the perioperative immune–inflammatory status with hypercoagulable states. In this retrospective study, a clinical prediction model for DVT during the perioperative period of arthroplasty was developed using LASSO regression and visualized by a nomogram. The nomogram involved seven predictive variables including age, chronic heart failure, stroke, tourniquet use, post-MLR, post-*α*, and post-D-dimer. Dividing the dataset based on the chronological order of surgery dates, as opposed to random sampling, more closely simulates true prospective prediction (33). The Brier score (0.1762 < 0.25), Hosmer–Lemeshow goodness-of-fit (*p* = 0.503 > 0.05), and AUC values (training set, 0.737; validation set, 0.683) demonstrated the model nomogram’s reasonable and robust predictive capability (34, 35). However, the calibration curve overestimated the risk in the higher range of postoperative DVT probability (>70%), potentially owing to the limited number of complex cases available for modeling. Given the strong correlation between immune-inflammatory and coagulation markers, one of these features tend to be selected in LASSO regression to manage multicollinearity and reduce model variance (36). Consequently, LASSO regression was chosen instead of the traditional stepwise regression to screen for risk factors in this study.

Postoperative DVT is a serious but preventable complication during and after hospitalization (37), closely associated with baseline characteristics of patients, such as advanced age and cardiovascular diseases (9, 13). Studies on these factors in patients undergoing surgery, under trauma, or with cancer have confirmed their significance in the development of DVT (38, 39). With advancing age, there is a gradual decline in vascular function and blood circulation (40). A history of stroke or chronic heart failure may result in reduced blood flow, elevated blood viscosity, impaired endothelial function, and damaged vascular walls, consequently increasing the risk of DVT (41–43). In addition, our study did not establish a correlation between BMI and DVT occurrence, possibly attributable to the relatively limited distribution of extreme BMIs in the Chinese population (44), or the encouragement for patients to lose weight preoperatively.

Tourniquet application is recognized as a significant surgical factor influencing postoperative DVT. It not only limits postoperative mobility and exacerbates pain but also markedly stimulates tissue factor release through ischemia–reperfusion injury, thereby disrupting the coagulation balance (45). Prolonged tourniquet compression may lead to vascular endothelial damage, further promoting tissue factor release and platelet aggregation, thus heightening the risk of thrombosis. In a prospective study by Huang et al., the incidence of DVT in TKA was significantly lower in the early tourniquet release group (4.6% [9/196]) compared to the late release group (12% [24/200]; OR 0.35, 95% CI 0.16–0.78, *p* = 0.008) (46). This may also contribute to the observed differences in DVT incidence between hip and knee arthroplasty.

The study shows that on the first postoperative day, MLR exhibits higher predictive potential than NLR, SII, AISI, and SIRI, consistent with the findings of Zhu et al. (20). The difference in predictive ability between NLR and SIRI may be related to the temporal changes in inflammatory dynamics, which are influenced by the primary onset and subacute or acute inflammatory states (19, 23). After surgery, the trend for NLR and SII usually shows an initial rise followed by a decline, peaking within 48 h postoperatively (47). The reduced predictive value of AISI and SII may be attributed to increased platelet activation and consumption caused by surgical bleeding and wound healing, leading to a reduction in the postoperative platelet count that requires time for recovery or reactive increase (21, 48). Although controversy remains regarding the peripheral blood-derived index with the highest predictive value (22), a dynamic and combined monitoring approach can improve diagnostic efficacy.

This study confirmed the predictive role of TEG in identifying DVT, underscoring its superiority over conventional coagulation function screening methods (49). The *α* angle reflects the rate of blood clot formation and is determined by fibrinogen and platelet interaction, but mainly reflects fibrinogen function in the early stages of coagulation. The larger α angle indicates hyperfunction of fibrinogen, which will directly enhance the fibrin crosslinking speed, leading to the acceleration of the coagulation cascade and the rapid formation of the fibrin network, which can jointly build a stable blood clot with platelets and promote thrombosis (50). Our results demonstrate that postoperative TEG holds predictive value for postoperative DVT, while preoperative TEG does not, aligning with previous findings (51). Brill et al. conducted a study involving 983 patients with trauma and found that the risk of DVT increased two-fold in patients with high coagulation status detected by TEG (52). In addition, some studies (53, 54), have suggested that maximum amplitude (MA) exhibits high sensitivity in diagnosing elevated coagulation status. MA reflects the maximum strength of clots formed through the interaction between fibrinogen and platelets via the GPIIb/IIIa receptor and is positively correlated with platelet count and fibrinogen levels (49). Consequently, the lack of significant sensitivity on postoperative day one may be attributed to surgical bleeding and fluid dilution.

D-dimer serves as a valuable but controversial biomarker for detecting DVT. Its utility is influenced by key variables, such as age and etiology (55, 56), leading to significant fluctuations among patients with surgical trauma, with two distinct peaks observed on postoperative days one and seven, often exceeding the conventional institution-set limit of 0.5 FEU μg/mL set (57). In this study, the AUC for D-dimer in diagnosing DVT was 0.6244, with an optimal cutoff value of 4.52 FEU μg/mL, indicating the stability of D-dimer levels on postoperative day one in predicting DVT, however, dynamic adjustments of the cutoff values are necessary based on temporal variations (58, 59).

This study collected various perioperative medical data before anticoagulation intervention to develop a nomogram prediction model that simply and accurately distinguishes high-risk patients. However, there are some limitations. First, as this is a single-center study, future multicenter research and ongoing data gathering are required to optimize the prediction model and increase the results’ generalizability. To further reduce the population’s heterogeneity, a more detailed subgroup study is required. Second, the validation dataset may be influenced by selection and time biases, hence we are constantly gathering data for prospective validation. Third, because of the brief hospital stay, we are intensifying follow-up to consider the impact of the time course of inflammatory markers on DVT risk and to account for DVT risk over longer timeframe. Finally, we established an association between the variables and DVT rather than a causal relationship, thus, requiring cautious interpretation of our results.

## Conclusion

In conclusion, age, chronic heart failure, stroke, tourniquet usage, post-MLR, post-*α*, and post-D-dimer are independent factors associated with predicting perioperative DVT in hip and knee arthroplasty. Based on these seven indicators, a predictive model was developed, which demonstrated good efficacy in predicting DVT risk. This model holds the potential to increase the clinical benefits for patients by identifying high-risk individuals early in the postoperative period.

## Data Availability

The raw data supporting the conclusions of this article will be made available by the authors, without undue reservation.

## Glossary

DVT: Deep vein thrombosis
LASSO: The Least Absolute Shrinkage and Selection Operator
AUC: Rea under the curve
DCA: Decision curve analysis
CIC: Clinical impact curves
TEG: Thromboelastography
CDUS: Complete duplex ultrasound
BMI: Body mass index
FEU: Fibrinogen equivalent units
ESR: Erythrocyte sedimentation rate
NLR: Neutrophil-to-lymphocyte ratio
MLR: Monocyte-to-lymphocyte ratio
PLR: Platelet-to-lymphocyte ratio
PNR: Platelet-to-neutrophil ratio
SII: Systemic immune-inflammation index
SIRI: Systemic immune response index
AISI: Aggregation index of systemic inflammation
MA: Maximum amplitude
Cl: Confidence interval
OR: Odds ratio
