# Supplementary material for: Development and validation of a nomogram prediction model for perioperative deep vein thrombosis risk in arthroplasty: a retrospective study
Source: Front Med (Lausanne). 2025 May 22;12:1528154. doi: 10.3389/fmed.2025.1528154 (PMC12137233; doi:10.3389/fmed.2025.1528154)
Supplement: Supplementary file 1 [file Table_1.docx]

Supplementary Table 1. Univariate analysis of non-DVT and DVT groups in all datasets.

| Variable | Training set(n = 621) | | | Validation set (n = 220) | | | All patients (n = 841) | |  |
| --- | --- | --- | --- | --- | --- | --- | --- | --- | --- |
|  | Non-DVT group(n =442) | DVT group(n = 179) | *P*-value | Non-DVT group(n =168) | DVT group(n = 52) | *P*-value | Non-DVT group(n =610) | DVT group(n = 231) | *P*-value |
| Sex (Female), n (%) | 282 (63.8) | 133 (74.3) | 0.012* | 122 (72.6) | 38 (73.1) | 0.948 | 404 (66.2) | 171 (74.0) | 0.03* |
| Age (years), n (%) |  |  | <0.001* |  |  | <0.001* |  |  | <0.001* |
| < 60 | 115 (26.0) | 20 (11.2) |  | 45 (26.8) | 2 (3.8) |  | 160 (26.2) | 22 (9.5) |  |
| 60–69 | 220 (49.8) | 92 (51.4) |  | 79 (47.0) | 22 (42.3) |  | 299 (49.0) | 114 (49.4) |  |
| ≥ 70 | 107 (24.2) | 67 (37.4) |  | 44 (26.2) | 28 (53.8) |  | 151 (24.8) | 95 (41.1) |  |
| BMI (≥28kg/m²), n (%) | 119 (26.9) | 48 (26.8) | 0.978 | 41 (24.4) | 12 (23.1) | 0.845 | 160 (26.2) | 60 (26.0) | 0.94 |
| Length of stay(days), (median [IQR]) | 8 [7, 9] | 7 [7, 9] | 0.639 | 7 [7, 9] | 7 [7, 8] | 0.125 | 8 [7, 9] | 7 [7, 9] | 0.291 |
| Smoker, n (%) | 71 (16.1) | 16 (8.9) | 0.021* | 18 (10.7) | 6 (11.5) | 0.868 | 89 (14.6) | 22 (9.5) | 0.053 |
| Drinker, n (%) | 61 (13.8) | 14 (7.8) | 0.053 | 17 (10.1) | 5 (9.6) | 0.916 | 78 (12.8) | 19 (8.2) | 0.065 |
| Hypertension, n (%) | 197 (44.6) | 99 (55.3) | 0.015* | 72 (42.9) | 29 (55.8) | 0.103 | 269 (44.1) | 128 (55.4) | 0.003* |
| Diabetes, n (%) | 51 (11.5) | 33 (18.4) | 0.023* | 23 (13.7) | 8 (15.4) | 0.759 | 74 (12.1) | 41 (17.7) | 0.034* |
| Chronic heart failure, n (%) | 30 (6.8) | 30 (16.8) | <0.001* | 14 (8.3) | 9 (17.3) | 0.065 | 44 (7.2) | 39 (16.9) | <0.001* |
| Hyperlipidemia, n (%) | 6 (1.4) | 3 (1.7) | 0.764 | 2 (1.2) | 3 (5.8) | 0.087 | 8 (1.3) | 6 (2.6) | 0.193 |
| Stroke, n (%) | 13 (2.9) | 15 (8.4) | 0.003* | 6 (3.6) | 10 (19.2) | <0.001* | 19 (3.1) | 25 (10.8) | <0.001* |
| Varicose vein, n (%) | 5 (1.1) | 2 (1.1) | 0.988 | 1 (0.6) | 1 (1.9) | 0.418 | 6 (1.0) | 3 (1.3) | 0.692 |
| History of blood transfusion, n (%) | 12 (2.7) | 4 (2.2) | 0.732 | 7 (4.2) | 2 (3.8) | 1 | 19 (3.1) | 6 (2.6) | 0.693 |
| Previous surgery, n (%) | 182 (41.2) | 85 (47.5) | 0.15 | 79 (47.0) | 28 (53.8) | 0.39 | 261 (42.8) | 113 (48.9) | 0.11 |
| Duration of surgery (min), (median [IQR]) | 95 [83, 112] | 90 [75, 109] | 0.009* | 90 [80, 110] | 90 [76, 102] | 0.433 | 94 [80, 110] | 90 [75, 109] | 0.008* |
| Mode of operation, n (%) |  |  | <0.001* |  |  | 0.482 |  |  | <0.001* |
| UKA | 84 (19.0) | 28 (15.6) |  | 38 (22.6) | 10 (19.2) |  | 122 (20.0) | 38 (16.5) |  |
| TKA | 224 (50.7) | 127 (70.9) |  | 81 (48.2) | 31 (59.6) |  | 305 (50.0) | 158 (68.4) |  |
| THA | 124 (28.1) | 16 (8.9) |  | 41 (24.4) | 10 (19.2) |  | 165 (27.0) | 26 (11.3) |  |
| HA | 10 (2.3) | 8 (4.5) |  | 8 (4.8) | 1 (1.9) |  | 18 (3.0) | 9 (3.9) |  |
| Protopathy, n (%) |  |  | <0.001* |  |  | 0.347 |  |  | <0.001* |
| OA | 305 (69.0) | 153 (85.5) |  | 122 (72.6) | 44 (84.6) |  | 427 (70.0) | 197 (85.3) |  |
| RA | 20 (4.5) | 3 (1.7) |  | 6 (3.6) | 1 (1.9) |  | 26 (4.3) | 4 (1.7) |  |
| AVN | 68 (15.4) | 7 (3.9) |  | 17 (10.1) | 4 (7.7) |  | 85 (13.9) | 11 (4.8) |  |
| DDH | 18 (4.1) | 8 (4.5) |  | 9 (5.4) | 0 (0.0) |  | 27 (4.4) | 8 (3.5) |  |
| FNF | 31 (7.0) | 8 (4.5) |  | 14 (8.3) | 3 (5.8) |  | 45 (7.4) | 11 (4.8) |  |
| Spinal anesthesia, n (%) | 415 (93.9) | 166 (92.7) | 0.596 | 166 (98.8) | 50 (96.2) | 0.38 | 581 (95.2) | 216 (93.5) | 0.312 |
| Air pressure therapy, n (%) | 130 (29.4) | 55 (30.7) | 0.746 | 39 (23.2) | 18 (34.6) | 0.101 | 169 (27.7) | 73 (31.6) | 0.265 |
| Intraoperative blood loss(≥200ml), n(%) | 125 (28.3) | 36 (20.1) | 0.035* | 27 (16.1) | 5 (9.6) | 0.249 | 152 (24.9) | 41 (17.7) | 0.027* |
| Tourniquet, n (%) | 288 (65.2) | 152 (84.9) | <0.001* | 119 (70.8) | 41 (78.8) | 0.257 | 407 (66.7) | 193 (83.5) | <0.001* |
| Intraoperative blood transfusion, n (%) | 56 (12.7) | 20 (11.2) | 0.606 | 13 (7.7) | 4 (7.7) | 1 | 69 (11.3) | 24 (10.4) | 0.704 |
| Preoperative laboratory examination | | | | | | | | | |
| Drainage tube, n (%) | 106 (24.0) | 50 (27.9) | 0.304 | 32 (19.0) | 14 (26.9) | 0.222 | 138 (22.6) | 64 (27.7) | 0.124 |
| Platelet count(*10⁹/L), (median [IQR]) | 230.00 [190.50, 269.75] | 232.00 [192.00, 262.50] | 0.901 | 228.00 [196.00, 281.00] | 231.00 [172.00, 267.50] | 0.401 | 230.00 [193.00, 273.00] | 232.00 [191.00, 263.50] | 0.548 |
| Lymphocyte count(*10⁹/L), (median [IQR]) | 1.80 [1.47, 2.23] | 1.80 [1.41, 2.20] | 0.815 | 1.85 [1.49, 2.30] | 1.77 [1.34, 2.12] | 0.179 | 1.82 [1.47, 2.25] | 1.79 [1.40, 2.20] | 0.349 |
| Monocyte count(*10⁹/L), (median [IQR]) | 0.43 [0.34, 0.52] | 0.43 [0.37, 0.52] | 0.181 | 0.41 [0.33, 0.51] | 0.39 [0.32, 0.45] | 0.179 | 0.43 [0.34, 0.52] | 0.42 [0.35, 0.51] | 0.579 |
| Neutrophil count(*10⁹/L), (median [IQR]) | 3.66 [2.88, 4.55] | 3.47 [2.86, 4.18] | 0.241 | 3.62 [3.02, 4.56] | 3.80 [2.95, 5.29] | 0.528 | 3.64 [2.90, 4.56] | 3.53 [2.86, 4.31] | 0.482 |
| NLR, (median [IQR]) | 1.99 [1.48, 2.58] | 1.89 [1.51, 2.44] | 0.45 | 1.97 [1.47, 2.62] | 2.34 [1.63, 3.04] | 0.065 | 1.98 [1.48, 2.59] | 1.97 [1.52, 2.59] | 0.706 |
| PLR, (median [IQR]) | 123.63 [101.23, 154.60] | 126.24 [101.91, 158.23] | 0.68 | 124.86 [96.70, 156.13] | 124.62 [100.51, 162.90] | 0.819 | 123.97 [100.00, 155.33] | 126.15 [101.34, 160.26] | 0.606 |
| MLR, (median [IQR]) | 0.23 [0.19, 0.30] | 0.23 [0.20, 0.31] | 0.167 | 0.21 [0.17, 0.27] | 0.21 [0.19, 0.28] | 0.724 | 0.23 [0.18, 0.29] | 0.23 [0.19, 0.31] | 0.13 |
| PNR, (median [IQR]) | 63.75 [50.14, 78.37] | 67.63 [52.48, 83.02] | 0.165 | 63.33 [49.05, 80.97] | 53.72 [44.28, 75.25] | 0.064 | 63.74 [49.57, 79.31] | 64.45 [49.43, 81.67] | 0.823 |
| SII, (median [IQR]) | 439.79 [319.78, 620.08] | 432.29 [339.12, 598.28] | 0.59 | 456.03 [320.09, 680.10] | 478.28 [330.48, 753.63] | 0.63 | 442.94 [319.78, 638.17] | 437.52 [335.76, 615.78] | 0.874 |
| SIRI, (median [IQR]) | 0.82 [0.59, 1.20] | 0.83 [0.61, 1.13] | 0.83 | 0.79 [0.54, 1.19] | 0.87 [0.60, 1.20] | 0.501 | 0.80[0.58, 1.19] | 0.84 [0.61, 1.14] | 0.564 |
| AISI, (median [IQR]) | 192.19 [123.03, 283.60] | 186.10 [129.26, 283.07] | 0.841 | 198.65 [114.07, 291.18] | 177.17 [113.47, 338.02] | 0.824 | 193.10 [120.83, 284.14] | 183.78 [127.18, 293.11] | 0.896 |
| R (min), (median [IQR]) | 6.20 [5.60, 6.80] | 6.20 [5.60, 6.80] | 0.807 | 5.80 [5.30, 6.43] | 5.90 [5.40, 6.73] | 0.471 | 6.00 [5.50, 6.80] | 6.20 [5.50, 6.80] | 0.456 |
| K (min), (median [IQR]) | 1.40 [1.20, 1.80] | 1.40 [1.20, 1.70] | 0.354 | 1.30 [1.20, 1.60] | 1.40 [1.20, 1.60] | 0.266 | 1.40 [1.20, 1.70] | 1.40 [1.20, 1.70] | 0.865 |
| α, (median [IQR]) | 68.05 [64.32, 70.70] | 68.20 [65.95, 70.80] | 0.152 | 68.70 [65.88, 70.93] | 67.70 [65.95, 70.25] | 0.411 | 68.30 [64.70, 70.70] | 68.20 [65.95, 70.60] | 0.43 |
| MA (mm), (median [IQR]) | 62.95 [59.40, 66.00] | 62.90 [60.05, 66.30] | 0.496 | 63.35 [60.08, 66.60] | 62.80 [59.48, 65.58] | 0.573 | 63.00 [59.60, 66.20] | 62.90 [59.90, 66.05] | 0.766 |
| CI, (median [IQR]) | 0.50 [-0.50, 1.37] | 0.50 [-0.20, 1.35] | 0.573 | 0.80 [0.00, 1.70] | 0.75 [-0.10, 1.50] | 0.509 | 0.60 [-0.40, 1.40] | 0.60 [-0.20, 1.40] | 0.972 |
| LY30%, (median [IQR]) | 0.00 [0.00, 0.00] | 0.00 [0.00, 0.00] | 0.217 | 0.00 [0.00, 0.00] | 0.00 [0.00, 0.00] | 0.851 | 0.00 [0.00, 0.00] | 0.00 [0.00, 0.00] | 0.34 |
| EPL%, (median [IQR]) | 0.00 [0.00, 0.00] | 0.00 [0.00, 0.00] | 0.306 | 0.00 [0.00, 0.00] | 0.00 [0.00, 0.00] | 0.851 | 0.00 [0.00, 0.00] | 0.00 [0.00, 0.00] | 0.424 |
| PT (s), (median [IQR]) | 12.36 [11.00, 13.60] | 12.70 [11.02, 13.60] | 0.584 | 10.90 [10.51, 11.50] | 10.80 [10.48, 11.39] | 0.543 | 11.57 [10.80, 13.28] | 11.87 [10.71, 13.40] | 0.352 |
| INR, (median [IQR]) | 0.97 [0.92, 1.02] | 0.97 [0.92, 1.02] | 0.812 | 0.96 [0.91, 1.00] | 0.95 [0.91, 0.98] | 0.421 | 0.96 [0.92, 1.02] | 0.96 [0.92, 1.02] | 0.994 |
| APTT (s), (median [IQR]) | 29.10 [27.38, 31.40] | 29.70 [27.29, 31.27] | 0.486 | 27.88 [25.83, 29.77] | 27.12 [25.79, 29.45] | 0.502 | 28.64 [26.92, 31.13] | 29.10 [26.86, 30.85] | 0.588 |
| TT (s), (median [IQR]) | 16.10 [15.10, 16.90] | 16.00 [15.10, 17.25] | 0.506 | 16.59 [15.66, 18.10] | 17.14 [15.89, 17.99] | 0.377 | 16.20 [15.27, 17.24] | 16.24 [15.32, 17.50] | 0.449 |
| D-dimer (ug/ml FEU), (median [IQR]) | 0.31 [0.20, 0.57] | 0.31 [0.22, 0.57] | 0.603 | 0.41 [0.24, 1.09] | 0.47 [0.27, 0.86] | 0.657 | 0.33 [0.21, 0.59] | 0.33 [0.22, 0.64] | 0.596 |
| CRP(mg/L), (median [IQR]) | 2.12 [1.20, 4.98] | 2.05 [1.25, 4.36] | 0.756 | 3.26 [1.93, 6.44] | 3.46 [1.89, 5.89] | 0.957 | 2.55 [1.35, 5.34] | 2.25 [1.33, 4.84] | 0.547 |
| ESR (mm/H) (median [IQR]) | 14.00 [7.00, 21.00] | 14.00 [8.00, 25.00] | 0.041* | 14.00 [9.00, 22.00] | 16.00 [8.00, 24.25] | 0.529 | 14.00 [8.00, 21.00] | 14.00 [8.00, 25.00] | 0.048* |
| Postoperative laboratory examination | | | | | | | | | |
| Platelet count(*10⁹/L), (median [IQR]) | 213.00 [180.00, 254.75] | 218.00 [177.00, 251.50] | 0.922 | 219.00 [190.75, 260.75] | 206.00 [171.25, 263.50] | 0.196 | 215.00 [182.00, 256.00] | 212.00 [175.00, 253.00] | 0.401 |
| Lymphocyte count(*10⁹/L), (median [IQR]) | 1.06 [0.80, 1.34] | 0.96 [0.76, 1.28] | 0.091 | 1.02 [0.76, 1.25] | 1.00 [0.75, 1.16] | 0.61 | 1.04 [0.79, 1.32] | 0.97 [0.75, 1.26] | 0.095 |
| Monocyte count(*10⁹/L) (median [IQR]) | 0.69 [0.53, 0.89] | 0.71 [0.56, 0.94] | 0.102 | 0.60 [0.45, 0.77] | 0.63 [0.48, 0.83] | 0.265 | 0.66 [0.51, 0.86] | 0.70 [0.55, 0.91] | 0.029* |
| Neutrophil count(*10⁹/L), (median [IQR]) | 9.08 [7.12, 11.07] | 9.11 [7.34, 11.59] | 0.377 | 8.86 [7.30, 11.20] | 8.94 [7.32, 11.12] | 0.927 | 8.98 [7.15, 11.13] | 9.08 [7.30, 11.51] | 0.472 |
| NLR, (median [IQR]) | 8.52 [6.33, 11.93] | 9.35 [6.61, 13.17] | 0.047* | 9.24 [6.12, 12.84] | 10.07 [7.22, 11.92] | 0.561 | 8.70 [6.22, 12.23] | 9.74 [6.66, 12.92] | 0.046* |
| PLR, (median [IQR]) | 204.43 [151.57, 269.62] | 224.72 [159.64, 283.06] | 0.101 | 214.14 [160.43, 287.93] | 214.80 [171.36, 284.61] | 0.697 | 207.78 [156.01, 275.25] | 221.33 [161.68, 283.17] | 0.254 |
| MLR, (median [IQR]) | 0.63 [0.49, 0.84] | 0.73 [0.51, 1.05] | 0.001* | 0.56 [0.43, 0.78] | 0.64 [0.52, 0.82] | 0.109 | 0.62 [0.47, 0.82] | 0.71 [0.52, 0.92] | <0.001* |
| PNR, (median [IQR]) | 23.95 [19.07, 29.45] | 23.80 [17.99, 29.19] | 0.422 | 25.15 [19.34, 31.35] | 22.60 [19.16, 28.42] | 0.122 | 24.14 [19.08, 29.97] | 23.34 [18.19, 29.07] | 0.149 |
| SII, (median [IQR]) | 1878.57 [1212.87, 2619.91] | 1994.81 [1305.45, 2901.62] | 0.111 | 2063.55 [1282.29, 2937.39] | 1986.52 [1386.66, 2803.22] | 0.828 | 1944.58 [1245.10, 2702.91] | 1992.92 [1312.86, 2892.90] | 0.244 |
| SIRI, (median [IQR]) | 5.55 [3.94, 8.30] | 6.73 [4.43, 10.22] | 0.003* | 5.04 [3.40, 7.34] | 6.08 [4.02, 8.10] | 0.142 | 5.48 [3.76, 8.23] | 6.53 [4.22, 9.30] | <0.001* |
| AISI, (median [IQR]) | 1208.93 [729.57, 2005.97] | 1403.97 [889.66, 2211.59] | 0.013* | 1085.74 [692.97, 1701.77] | 1235.23 [802.58, 1702.87] | 0.425 | 1180.44 [725.21, 1926.63] | 1337.09 [877.87, 2048.96] | 0.011* |
| R (min), (median [IQR]) | 5.80 [5.20, 6.40] | 5.80 [5.20, 6.30] | 0.296 | 5.70 [5.20, 6.20] | 5.80 [5.20, 6.30] | 0.439 | 5.80 [5.20, 6.30] | 5.80 [5.20, 6.30] | 0.621 |
| K (min), (median [IQR]) | 1.30 [1.20, 1.60] | 1.20 [1.10, 1.50] | 0.026* | 1.20 [1.20, 1.40] | 1.20 [1.17, 1.50] | 0.945 | 1.30 [1.20, 1.50] | 1.20 [1.10, 1.50] | 0.057 |
| α, (median [IQR]) | 69.20 [66.53, 71.80] | 70.00 [67.80, 72.30] | 0.019* | 69.30 [67.68, 71.23] | 69.45 [67.02, 71.62] | 0.924 | 69.20 [66.80, 71.60] | 69.80 [67.45, 72.10] | 0.036* |
| MA (mm),(median [IQR]) | 64.15 [60.90, 67.30] | 64.30 [61.40, 67.00] | 0.563 | 63.70 [61.20, 66.75] | 63.55 [60.72, 66.53] | 0.793 | 64.10 [60.92, 67.20] | 64.20 [61.20, 67.00] | 0.659 |
| CI, (median [IQR]) | 1.00 [0.20, 1.80] | 1.10 [0.40, 1.70] | 0.166 | 1.00 [0.40, 1.70] | 0.90 [0.20, 1.75] | 0.646 | 1.00 [0.20, 1.80] | 1.10 [0.35, 1.70] | 0.317 |
| LY30%, (median [IQR]) | 0.00 [0.00, 0.00] | 0.00 [0.00, 0.00] | 0.818 | 0.00 [0.00, 0.00] | 0.00 [0.00, 0.00] | 0.578 | 0.00 [0.00, 0.00] | 0.00 [0.00, 0.00] | 0.729 |
| EPL%, (median [IQR]) | 0.00 [0.00, 0.00] | 0.00 [0.00, 0.00] | 0.306 | 0.00 [0.00, 0.00] | 0.00 [0.00, 0.00] | 0.578 | 0.00 [0.00, 0.00] | 0.00 [0.00, 0.00] | 0.269 |
| PT (s), (median [IQR]) | 12.85 [11.67, 14.20] | 12.59 [11.63, 14.23] | 0.507 | 11.70 [11.12, 12.28] | 11.66 [11.12, 12.18] | 0.846 | 12.34 [11.50, 13.73] | 12.20 [11.52, 13.81] | 0.803 |
| INR, (median [IQR]) | 1.04 [0.99, 1.10] | 1.04 [0.99, 1.09] | 0.662 | 1.03 [0.98, 1.09] | 1.04 [0.96, 1.08] | 0.436 | 1.03 [0.98, 1.10] | 1.04 [0.99, 1.09] | 0.955 |
| APTT (s), (median [IQR]) | 28.70 [26.80, 30.70] | 28.99 [27.40, 31.05] | 0.216 | 27.84 [26.32, 29.55] | 28.15 [27.10, 29.30] | 0.589 | 28.52 [26.64, 30.48] | 28.73 [27.30, 30.70] | 0.143 |
| TT (s), (median [IQR]) | 15.70 [14.65, 16.68] | 15.88 [14.90, 16.80] | 0.269 | 15.98 [15.02, 16.83] | 15.79 [14.83, 16.80] | 0.682 | 15.75 [14.80, 16.70] | 15.87 [14.90, 16.80] | 0.514 |
| D-dimer (ug/ml FEU), (median [IQR]) | 2.75 [1.61, 5.52] | 4.58 [2.15, 7.47] | <0.001* | 3.98 [2.04, 6.24] | 3.26 [1.63, 7.46] | 0.813 | 3.07 [1.70, 5.80] | 4.30 [2.04, 7.46] | <0.001* |

AVN, avascular necrosis; BMI, body mass index; DDH, developmental dysplasia of the hip; FNF, fracture of neck of femur; IQR, interquartile range; OA, osteoarthritis; DVT, deep venous thrombosis; HA, hemiarthroplasty; RA, rheumatic arthritis; THA, total hip arthroplasty; TKA, total knee arthroplasty; UKA, unicompartmental knee arthroplasty; FEU, fibrinogen equivalent units; CRP, C-reactive protein; ESR, erythrocyte sedimentation rate; NLR, neutrophil-to-lymphocyte ratio; MLR, monocyte-to-lymphocyte ratio; PLR, platelet-to-lymphocyte ratio; PNR, platelet-to-neutrophil ratio; SII, systemic immune-inflammation index; SIRI, systemic immune response index; AISI, systemic inflammation aggregation index; R, reaction time; K, k value; α, alpha angle; MA, maximum amplitude; CI, coagulation index; PT, prothrombin time; INR, International Normalized Ratio; APTT, activated partial thromboplastin time; TT, thrombin time.

*Significant difference (P<0.05)
