# Supplementary material for: Development and validation of a nomogram prediction model for perioperative deep vein thrombosis risk in arthroplasty: a retrospective study
Source: Front Med (Lausanne). 2025 May 22;12:1528154. doi: 10.3389/fmed.2025.1528154 (PMC12137233; doi:10.3389/fmed.2025.1528154)
Supplement: Supplementary file 2 [file Table_2.docx]

Supplementary Table 2, Sub-analyses of hip or knee replacement in the training dataset.

| Variable | knee replacement surgery (n=463) | | | Hip replacement surgery (n=158) | | |
| --- | --- | --- | --- | --- | --- | --- |
|  | non-DVT (n =308) | DVT (n =155) | *P*-value | non-DVT (n =134) | DVT (n =24) | *P*-value |
| Sex (Female), n (%) | 208 (67.5) | 113 (72.9) | 0.237 | 74 (55.2) | 20 (83.3) | 0.01 |
| Age (years), n (%) |  |  | 0.058* |  |  | 0.007* |
| < 60 | 50 (16.2) | 16 (10.3) |  | 65 (48.5) | 4 (16.7) |  |
| 60 – 69 | 170 (55.2) | 80 (51.6) |  | 50 (37.3) | 12 (50.0) |  |
| ≥ 70 | 88 (28.6) | 59 (38.1) |  | 19 (14.2) | 8 (33.3) |  |
| BMI (≥28kg/m²), n (%) | 102 (33.1) | 45 (29.0) | 0.373 | 17 (12.7) | 3 (12.5) | 0.98 |
| Length of stay (days), (median [IQR]) | 8.00 [7.00, 9.00] | 8.00 [7.00, 9.00] | 0.815 | 8.00 [7.00, 10.00] | 7.00 [6.00, 9.00] | 0.171 |
| Smoker, n (%) | 40 (13.0) | 15 (9.7) | 0.299 | 31 (23.1) | 1 (4.2) | 0.033* |
| Drinker, n (%) | 31 (10.1) | 12 (7.7) | 0.416 | 30 (22.4) | 2 (8.3) | 0.115 |
| Hypertension, n (%) | 154 (50.0) | 92 (59.4) | 0.057 | 43 (32.1) | 7 (29.2) | 0.777 |
| Diabetes, n (%) | 39 (12.7) | 32 (20.6) | 0.024 | 12 (9.0) | 1 (4.2) | 0.432 |
| Chronic heart failure, n (%) | 23 (7.5) | 25 (16.1) | 0.004* | 7 (5.2) | 5 (20.8) | 0.008* |
| Hyperlipidemia, n (%) | 4 (1.3) | 3 (1.9) | 0.596 | 2 (1.5) | 0 (0.0) | 0.547 |
| Stroke, n (%) | 8 (2.6) | 12 (7.7) | 0.01* | 5 (3.7) | 3 (12.5) | 0.071 |
| Varicose vein, n (%) | 5 (1.6) | 2 (1.3) | 0.782 |  |  |  |
| History of blood transfusion, n (%) | 7 (2.3) | 3 (1.9) | 0.814 | 5 (3.7) | 1 (4.2) | 0.918 |
| Previous surgery, n (%) | 119 (38.6) | 73 (47.1) | 0.081 | 63 (47.0) | 12 (50.0) | 0.787 |
| Duration of surgery (min), (median [IQR]) | 95.00 [84.00, 110.00] | 90.00 [75.00, 109.00] | 0.016* | 99.00 [81.50, 123.50] | 100.00 [73.00, 119.75] | 0.504 |
| Mode of operation, n (%) |  |  | 0.029* |  |  | <0.001* |
| UKA | 84 (27.3) | 28 (18.1) |  |  |  |  |
| TKA | 224 (72.7) | 127 (81.9) |  |  |  |  |
| THA |  |  |  | 124 (92.5) | 16 (66.7) | <0.001 |
| HA |  |  |  | 10 (7.5) | 8 (33.3) |  |
| Protopathy, n (%) |  |  | 0.587 |  |  | 0.04* |
| OA | 291 (94.5) | 151 (97.4) |  | 14 (10.4) | 2 (8.3) |  |
| RA | 12 (3.9) | 3 (1.9) |  | 8 (6.0) | 0 (0.0) |  |
| AVN | 1 (0.3) | 0 (0.0) |  | 67 (50.0) | 7 (29.2) |  |
| DDH | 2 (0.6) | 0 (0.0) |  | 16 (11.9) | 8 (33.3) |  |
| FNF | 2 (0.6) | 1 (0.6) |  | 29 (21.6) | 7 (29.2) |  |
| Spinal anesthesia, n (%) | 13 (4.2) | 11 (7.1) | 0.188 | 14 (10.4) | 2 (8.3) | 0.752 |
| Air pressure therapy, n (%) | 92 (29.9) | 50 (32.3) | 0.599 | 38 (28.4) | 5 (20.8) | 0.446 |
| Intraoperative blood loss(≥200ml), n(%) | 44 (14.3) | 24 (15.5) | 0.731 | 81 (60.4) | 12 (50.0) | 0.338 |
| Tourniquet, n (%) | 288 (93.5) | 152 (98.1) | 0.033 |  |  |  |
| Intraoperative blood transfusion, n (%) | 14 (4.5) | 11 (7.1) | 0.252 | 42 (31.3) | 9 (37.5) | 0.552 |
| Drainage tube, n (%) | 83 (26.9) | 41 (26.5) | 0.909 | 23 (17.2) | 9 (37.5) | 0.022* |
| Preoperative laboratory examination | | | | | | |
| Platelet count (*10⁹/L), (median [IQR])/(mean (SD)) | 229.00 [192.00, 265.25] | 233.00 [196.00, 260.50] | 0.614 | 238.19 (64.83) | 225.75 (63.95) | 0.387 |
| Lymphocyte count(*10⁹/L), (median [IQR]) | 1.83 [1.51, 2.25] | 1.85 [1.48, 2.22] | 0.971 | 1.75 [1.33, 2.18] | 1.49 [1.19, 2.04] | 0.14 |
| Monocyte count(*10⁹/L), (median [IQR]) | 0.43 [0.34, 0.51] | 0.43 [0.36, 0.52] | 0.183 | 0.43 [0.35, 0.54] | 0.46 [0.37, 0.52] | 0.613 |
| Neutrophil count(*10⁹/L), (median [IQR]) | 3.54 [2.82, 4.38] | 3.49 [2.91, 4.10] | 0.906 | 3.90 [2.97, 4.98] | 3.30 [2.59, 4.42] | 0.225 |
| NLR, (median [IQR]) | 1.94 [1.44, 2.44] | 1.88 [1.50, 2.34] | 0.92 | 2.24 [1.63, 2.98] | 2.30 [1.58, 3.24] | 0.904 |
| PLR, (median [IQR]) | 121.24 [99.49, 152.12] | 122.18 [100.23, 156.92] | 0.665 | 135.36 [104.59, 166.51] | 150.21 [121.02, 177.55] | 0.181 |
| MLR, (median [IQR]) | 0.23 [0.18, 0.29] | 0.23 [0.20, 0.31] | 0.213 | 0.23 [0.19, 0.31] | 0.25 [0.22, 0.38] | 0.122 |
| PNR, (median [IQR]) | 64.24 [52.31, 78.08] | 67.63 [52.48, 80.34] | 0.508 | 61.93 [45.74, 78.64] | 68.13 [52.75, 90.87] | 0.3 |
| SII, (median [IQR]) | 424.81 [313.40, 563.11] | 415.80 [339.12, 551.99] | 0.859 | 493.03 [359.98, 747.73] | 452.25 [345.24, 776.91] | 0.965 |
| SIRI, (median [IQR]) | 0.79 [0.56, 1.12] | 0,83 [0.61, 1.11] | 0.492 | 0.87 [0.66,1.39 ] | 0.98 [0.53, 1.45] | 0.988 |
| AISI, (median [IQR]) | 185.33 [117.08, 272.26] | 183.61 [129.26, 260.82] | 0.425 | 218.06 [138.58, 336.34] | 222.95 [128.77, 389.31] | 0.85 |
| R (min), (median [IQR])/(mean (SD)) | 6.20 [5.60, 6.80] | 6.10 [5.50, 6.80] | 0.721 | 6.18 (1.19) | 6.53 (0.87) | 0.179 |
| K (min), (median [IQR]) | 1.40 [1.20, 1.80] | 1.40 [1.20, 1.70] | 0.606 | 1.40 [1.20, 1.80] | 1.45 [1.20, 1.60] | 0.369 |
| α (median [IQR]) | 68.30 [64.57, 70.50] | 68.20 [65.55, 70.95] | 0.309 | 67.95 [63.80, 70.77] | 68.95 [66.30, 70.45] | 0.294 |
| MA (mm), (median [IQR]) | 63.00 [59.90, 65.70] | 62.80 [59.90, 65.60] | 0.952 | 62.65 [58.82, 66.20] | 63.95 [61.47, 66.88] | 0.173 |
| CI, (median [IQR]) | 0.55 [-0.50, 1.30] | 0.60 [-0.20, 1.35] | 0.744 | 0.40 [-0.60, 1.40] | 0.35 [-0.20, 1.27] | 0.782 |
| LY30%, (median [IQR]) | 0.00 [0.00, 0.00] | 0.00 [0.00, 0.00] | 0.697 | 0.00 [0.00, 0.00] | 0.00 [0.00, 0.00] | 0.168 |
| EPL%, (median [IQR]) | 0.00 [0.00, 0.00] | 0.00 [0.00, 0.00] | 0.962 | 0.00 [0.00, 0.00] | 0.00 [0.00, 0.00] | 0.168 |
| PT (s), (median [IQR]) | 12.20 [10.86, 13.53] | 12.60 [11.00, 13.60] | 0.341 | 12.63 [11.29, 13.80] | 13.00 [11.15, 13.65] | 0.981 |
| INR, (median [IQR]) | 0.96 [0.91, 1.01] | 0.97 [0.92, 1.02] | 0.268 | 0.99 [0.94, 1.05] | 0.96 [0.93, 1.05] | 0.694 |
| APTT (s), (median [IQR]) | 29.05 [27.43, 31.31] | 29.60 [27.13, 31.37] | 0.677 | 29.20 [27.33, 31.78] | 29.90 [28.58, 30.95] | 0.337 |
| TT (s), (median [IQR])/(mean (SD)) | 16.09 [15.10, 17.03] | 16.17 [15.20, 17.25] | 0.55 | 16.03 (1.33) | 16.10 (1.68) | 0.807 |
| D-dimer (ug/ml FEU), (median [IQR]) | 0.28 [0.20, 0.48] | 0.28 [0.19, 0.53] | 0.776 | 0.39 [0.24, 0.79] | 0.76 [0.40, 1.43] | 0.005* |
| CRP(mg/L), (median [IQR]) | 2.02 [1.06, 4.14] | 2.01 [1.23, 4.04] | 0.886 | 2.64 [1.41, 6.66] | 4.00 [1.58, 27.19] | 0.196 |
| ESR (mm/H), (median [IQR]) | 13.50 [8.00, 20.00] | 14.00 [8.00, 23.50] | 0.113 | 14.00 [7.00, 23.75] | 19.50 [12.00, 35.00] | 0.05* |
| Postoperative laboratory examination | | | | | | |
| Platelet count(*10⁹/L), (median [IQR]) | 216.00 [184.00, 256.00] | 219.00 [182.00, 252.00] | 0.83 | 206.50 [175.25, 249.50] | 197.00 [151.00, 247.00] | 0.502 |
| Lymphocyte count(*10⁹/L), (median [IQR]) | 1.03 [0.79, 1.34] | 0.94 [0.76, 1.28] | 0.226 | 1.14 [0.85, 1.33] | 1.10 [0.66, 1.29] | 0.443 |
| Monocyte count(*10⁹/L), (median [IQR]) | 0.66 [0.51, 0.86] | 0.70 [0.56, 0.95] | 0.031* | 0.76 [0.59, 0.94] | 0.74 [0.59, 0.91] | 0.913 |
| Neutrophil count(*10⁹/L), (median [IQR]) | 9.42 [7.33, 11.55] | 9.37 [7.54, 11.73] | 0.761 | 8.37 [6.58, 10.06] | 7.30 [6.22, 10.36] | 0.656 |
| NLR, (median [IQR]) | 9.21 [6.72, 12.79] | 9.85 [6.64, 13.30] | 0.315 | 7.18 [5.65, 10.01] | 7.47 [5.95, 12.32] | 0.532 |
| PLR, (median [IQR]) | 210.96 [153.96, 280.05] | 230.63 [167.76, 287.95] | 0.287 | 194.85 [148.96, 242.59] | 181.25 [154.12, 264.17] | 0.673 |
| MLR, (median [IQR]) | 0.62 [0.47, 0.83] | 0.72 [0.51, 0.99] | 0.002* | 0.63 [0.54, 0.85] | 0.76 [0.54, 1.15] | 0.102 |
| PNR, (median [IQR]) | 23.62 [18.77, 28.60] | 23.80 [17.91, 28.97] | 0.786 | 24.86 [20.30, 31.59] | 23.13 [19.45, 33.40] | 0.713 |
| SII, (median [IQR]) | 2037.14 [1331.80, 2831.09] | 2079.43 [1325.16, 2923.58] | 0.478 | 1537.34 [1023.56, 2276.84] | 1614.00 [1067.92, 2070.96] | 0.782 |
| SIRI, (median [IQR]) | 5.60 [3.96, 8.61] | 6,72 [4.51, 11.09] | 0.016* | 5.26 [3.68, 7.73] | 6.70 [3.40, 8.87] | 0.269 |
| AISI, (median [IQR]) | 1266.31 [791.00, 2078.44] | 1456.89 [903.33, 2263.21] | 0.057 | 1118.75 [657.71, 1714.32] | 1253.00 [715.08, 2038.35] | 0.51 |
| R (min), (median [IQR]) | 5.90 [5.20, 6.50] | 5.80 [5.20, 6.40] | 0.306 | 5.80 [5.12, 6.20] | 5.60 [5.20, 5.88] | 0.394 |
| K (min), (median [IQR]) | 1.30 [1.20, 1.60] | 1.20 [1.10, 1.50] | 0.033* | 1.30 [1.12, 1.60] | 1.20 [1.10, 1.42] | 0.257 |
| α, (median [IQR]) | 69.05 [66.20, 71.70] | 69.90 [67.35, 72.15] | 0.043* | 69.45 [66.80, 71.97] | 70.85 [68.38, 72.68] | 0.096 |
| MA (mm), (median [IQR])/(mean (SD)) | 64.07 (4.42) | 64.21 (4.09) | 0.749 | 64.02 (4.86) | 65.11 (4.73) | 0.31 |
| CI (median [IQR]) | 0.90 [0.20, 1.70] | 1.00 [0.30, 1.70] | 0.229 | 1.10 [0.00, 2.00] | 1.25 [0.75, 2.20] | 0.229 |
| LY30%, (median [IQR]) | 0.00 [0.00, 0.00] | 0.00 [0.00, 0.00] | 0.997 | 0.00 [0.00, 0.00] | 0.00 [0.00, 0.00] | 0.548 |
| EPL%, (median [IQR]) | 0.00 [0.00, 0.00] | 0.00 [0.00, 0.00] | 0.381 | 0.00 [0.00, 0.00] | 0.00 [0.00, 0.00] | 0.548 |
| PT (s), (median [IQR])/(mean (SD)) | 12.75 [11.56, 14.20] | 12.48 [11.62, 14.10] | 0.721 | 13.15 (1.57) | 13.33 (1.97) | 0.622 |
| INR, (median [IQR])/(mean (SD)) | 1.02 [0.98, 1.09] | 1.04 [0.99, 1.09] | 0.134 | 1.06 (0.09) | 1.05 (0.08) | 0.574 |
| APTT (s), (median [IQR]) | 28.51 [26.87, 30.52] | 28.90 [27.30, 31.14] | 0.222 | 28.99 [26.40, 30.89] | 29.67 [27.95, 30.84] | 0.383 |
| TT (s), (median [IQR])/(mean (SD)) | 15.58 [14.60, 16.52] | 15.88 [14.90, 16.77] | 0.118 | 15.87 (1.49) | 15.85 (1.50) | 0.954 |
| D-dimer (ug/ml FEU), (median [IQR]) | 2.68 [1.56, 5.07] | 4.59 [2.14, 7.63] | <0.001* | 2.80 [1.66, 6.02] | 4.25 [2.28, 7.06] | 0.207 |

AVN, avascular necrosis; BMI, body mass index; DDH, developmental dysplasia of the hip; FNF, fracture of neck of femur; IQR, interquartile range; OA, osteoarthritis; DVT, deep venous thrombosis; HA, hemiarthroplasty; RA, rheumatic arthritis; THA, total hip arthroplasty; TKA, total knee arthroplasty; UKA, unicompartmental knee arthroplasty; FEU, fibrinogen equivalent units; CRP, C-reactive protein; ESR, erythrocyte sedimentation rate; NLR, neutrophil-to-lymphocyte ratio; MLR, monocyte-to-lymphocyte ratio; PLR, platelet-to-lymphocyte ratio; PNR, platelet-to-neutrophil ratio; SII, systemic immune-inflammation index; SIRI, systemic immune response index; AISI, systemic inflammation aggregation index; R, reaction time; K, k value; α, alpha angle; MA, maximum amplitude; CI, coagulation index; PT, prothrombin time; INR, International Normalized Ratio; APTT, activated partial thromboplastin time; TT, thrombin time.

*Significant difference (P<0.05)
